# Mindful Opportunity to Reflect on Experience: Interdisciplinary Mind–Body Medicine Skills Training for Health-care Professionals

**DOI:** 10.1177/2164956120907876

**Published:** 2020-02-14

**Authors:** Jessica L Barnhill, Jonathan S Gerkin, Vera L Moura, Amy B Weil

**Affiliations:** 1 Department of Physical Medicine and Rehabilitation, University of North Carolina at Chapel Hill, Chapel Hill, North Carolina; 2 Department of Psychiatry, University of North Carolina at Chapel Hill, Chapel Hill, North Carolina; 3 Division of General Internal Medicine and Epidemiology, Department of Internal Medicine, University of North Carolina at Chapel Hill, Chapel Hill, North Carolina

**Keywords:** mind–body medicine, burnout, mindfulness, employee wellness, well-being

## Abstract

Interventions that support employee wellness and resilience hold potential to improve patient care, increase staff engagement, and decrease burnout. This repeat-measures study evaluated whether an abbreviated version of mind–body medicine skills training could decrease stress and improve mindfulness among an interdisciplinary cohort of health-care professionals. The study also assessed whether participants incorporated the mind–body medicine skills into their personal and professional lives. Aggregate results from this unpaired cohort showed decreased stress and increased mindfulness. Postcourse surveys demonstrated increased personal and professional use of mind–body medicine skills. There was high favorability among participants. These preliminary results suggest that a modest investment of time and resources to learn mind–body medicine skills may positively affect employee wellness among health-care professionals. In addition, skills learned could translate into improved patient care and increased staff engagement. Further study with larger cohorts and a paired design is needed.

## Introduction

As health-care organizations look to support the health and well-being of their employees, there is a need for evidence-based wellness interventions tailored to the health-care workplace. Interventions that teach employees new skills have the potential to improve their personal wellness while also equipping them with new clinical knowledge to enhance patient wellness.

This study tested the hypothesis that an abbreviated version of mind–body medicine skills training would reduce stress and increase mindfulness among a heterogeneous group of health-care professionals. Measures of personal and professional use of mind–body medicine skills were also assessed. These findings contribute to the growing body of literature on the adaptation of mind–body medicine skills training to improve well-being and to increase knowledge of these techniques in the health-care workforce.

While this intervention did not measure changes in levels of burnout, the existence of high levels of burnout in the health-care workforce was a motivating factor in the creation of this intervention. Burnout has been defined as

a psychological syndrome emerging as a prolonged response to chronic interpersonal stressors on the job. The three key dimensions of this response are an overwhelming exhaustion, feelings of cynicism and detachment from the job, and a sense of ineffectiveness and lack of accomplishment.^1^

Rates of burnout among health-care professionals are high, with more than half of all physicians affected by burnout.^2^

The link between an unhealthy workforce and inadequate patient care is well documented.^3–7^ For example, a review by Braun et al. found strong evidence that health-care professionals with higher mindfulness scores reported greater improvements in patient care.^4^ According to a meta-analysis by Salyers et al., “provider burnout shows consistent negative relationships with perceived quality (including patient satisfaction), quality indicators, and perceptions of safety.”^6^

Given the extent of burnout among health-care professionals and its effect on quality of care, interest in systemic reform and support for employee wellness has increased. A meta-analysis by Panagioti et al. found that burnout interventions had a small impact on burnout rates and that this impact was augmented by system-wide approaches. They conclude “burnout is a problem of the whole healthcare organization, rather than individuals.”^8^ In addition, Branch et al. have reported on the interconnectedness of organizational and personal domains in building and maintaining well-being among health-care professionals.^9^

A growing body of literature demonstrates that mindfulness-based interventions, including mind–body medicine skills, support the well-being of health-care professionals.^10^ Mindfulness practices such as meditation are known to balance the autonomic nervous system and promote neurophysiologic changes in the hypothalamic–pituitary axis that among other effects also enhance focus and calm.^11,12^ A systematic review by van der Riet et al. concluded that mindfulness interventions improve indicators of well-being of nurses and nursing students.^13^ Another systematic review by Dharmawardene et al. concluded that “Meditation provides a small to moderate benefit for informal caregivers and health professionals for stress reduction, but more research is required to establish effects on burnout and caregiver burden.”^14^

With regard to mind–body medicine skills, which are a subset of mindful practices, the work of Staples and Gordon has demonstrated improved wellness among health-care professionals.^15^ They surveyed 451 professionals who completed the 7-day course in mind–body medicine skills. Their results demonstrated higher life satisfaction scores. In addition, at 1-year follow-up, participants reported increased use of mind–body medicine skills in personal and professional life. Among medical students at Georgetown University, students who attended a course in mind–body medicine skills had decreased physiologic markers of stress as compared to the control group.^16^ Greeson et al. created an adapted version of the mind–body medicine skills course and trained medical students over a period of 4 sessions. Their results suggest that a brief course in mind–body medicine skills can improve well-being among medical students.^17^ This conclusion is consistent with the finding that mindfulness programs as short as 4 hours have demonstrated efficacy in health-care settings.^18^

Building upon this research base, this study describes an adapted mind–body medicine skills course tailored to the work environment and inclusive of a wide range of health-care professionals. The goal of the course, Mindful Opportunity to Reflect on Experience (MORE), was to support health-care professionals’ wellness while equipping them with skills to support their colleagues and patients. It was hypothesized that the course would decrease stress, increase mindfulness, and result in increased personal and professional use of mind–body medicine techniques.

## Materials

Faculty at the University of North Carolina at Chapel Hill School of Medicine received training in mind–body medicine skills at the Institute of Integrative Health. This curriculum was tailored, shortening the length and number of sessions, to accommodate the schedules of health-care professionals at the University of North Carolina at Chapel Hill School of Medicine. In consultation with trainers from Georgetown University, the number of sessions was decreased from 11 to 8, and some sessions were shortened from 120 minutes to 90 minutes (see Table 1 for a comparison of the original course and the tailored version).

**Table 1.** Comparison of Mind–Body Medicine Skills Course and Mindful Opportunity to Reflect on Experience

Title	Mind–Body Medicine Skills Course	Mindful Opportunity to Reflect on Experience
Institution	Georgetown University	University of North Carolina at Chapel Hill
Population	Medical students	Health-care professionals
Number of sessions	11 weeks	8 weeks
Length of session	120 min per session	120 min Cohort 1 90 min Cohort 2
Session 1	Opening meditation, drawings Part 1	Welcome and drawing
Session 2	Autogenic training/biofeedback	Mindful eating and mindfulness meditation
Session 3	Eating meditation, sitting mindfulness meditation	Autogenic training
Session 4	*Special place imagery* ^a^	Inner guide (guided imagery)
Session 5	Inner guide/wise self/spirit guide imagery	*Journal writing* ^a^
Session 6	*Dialogue with a symptom, body part, or emotion* ^a^	Forgiveness meditation
Session 7	*Shaking and dancing* ^a^	*Loving kindness meditation (and recitation)* ^a^
Session 8	Forgiveness meditation	Sharing positive quality cards, drawings Part 2, and closing ritual
Session 9	*Walking meditation* ^a^	
Session 10	Drawings Part 2, positive quality index cards	
Session 11	Closing ritual	

^a^Divergent course content.

## Methods

Faculty taught the course twice and drew participants from across health-care disciplines. These disciplines included Internal Medicine, Psychiatry, Emergency Medicine, Obstetrics and Gynecology, Speech and Hearing, Psychology, Library Science, Social Work, and Physician Assistants. To keep stress low, and decrease interference with work and home life, the course was scheduled at the end of the workday, from 4 pm to 6 pm for the first cohort and from 4 pm to 5:30 pm for the second cohort.

To measure the feasibility and efficacy of the course, instructors utilized repeat measures and short answer responses. The measures were independently completed twice, at the beginning and end of each course. Participants anonymously completed the Freiburg Mindfulness Inventory (FMI) survey, the Perceived Stress Scale (PSS), and a general survey regarding the personal and professional use of mind–body medicine skills. Nonclinical participants were instructed to not respond to questions regarding clinical mind–body medicine use. Postcourse evaluations included scored and short answer responses. The general survey was adapted from materials originating from the Center for Mind-Body Medicine.

These measures were chosen because they are the same measures reported by Greeson et al. in their study of mind–body medicine skills training among medical students.^17^ The short form of the FMI consists of 14 questions and higher scores correlate with increased mindfulness. Walach et al. found the short form of the FMI to be “semantically robust and psychometrically stable.”^19^ Similarly, Taylor reports that “findings suggest that inferences made using the PSS-10 are valid.”^20^

Among the first cohort, 9 participants enrolled and 8 completed the course. In the second cohort, 6 participants enrolled and 5 completed the course. In total, 15 participants have enrolled, and 13 participants have completed the course. This study was exempt from institutional review board approval because participant demographics were not collected, and measures were collected anonymously. Therefore, information on attrition patterns is not available. In addition, the following results were calculated by means of an unpaired *t* test with equal variance as opposed to a paired *t* test, and descriptive data comparing the first and second cohort are not available.

## Results

Mindfulness increased and stress decreased among course participants. The general survey showed increased utilization of mind–body medicine modalities. There was strong agreement with plans to teach these skills to colleagues and patients. Given the small unpaired sample, these trends were not statistically meaningful.

The precourse FMI mean score was 35.7 with a standard deviation of 6.7 and a 95% confidence interval of 32.0 to 39.5. The postcourse FMI mean score was 41.8 with a standard deviation of 5.0 and a 95% confidence interval of 38.6 to 44.9. The increase in FMI mean scores pre- and postcourse was 6.0 with a 95% confidence interval of 1.2 to 10.8.

The precourse PSS mean score was 18.2 with a standard deviation of 1.5 and a 95% confidence interval of 15.1 to 21.3. The postcourse PSS mean score was 14.2 with a standard deviation of 1.4 and a 95% confidence interval of 11.2 to 17.1. The change in PSS mean scores pre- and postcourse was −4.0 with a 95% confidence interval of −8.2 to 0.1.

In keeping with earlier research,^15,17^ prior training in mind–body medicine skills was assessed across 5 domains: biofeedback, imagery, meditation, exercise/movement, and psychosocial support. Among participants, the percentage with previous training in biofeedback was low at 6.7%. Participants were more familiar with the other modalities, reporting prior training in imagery at 26.7%, meditation at 73.3%, exercise/movement at 60.0%, and participation in psychosocial support groups at 33.3%.

Learners reported increased use of mind–body medicine skills in personal and professional life by the end of course. Of note, personal use of biofeedback increased 69.2%, and personal use of imagery increased 44.6% (see Table 2). Some class participants were not directly responsible for patient care, and they were not included in the assessment of clinical use. Although increases in use of mind–body medicine skills were present, the small sample size and aggregate analysis preclude drawing further conclusions from these data.

**Table 2.** Use of Mind–Body Medicine Modalities Pre- and Post-MORE Course.

	Percentage of participants with prior training (n = 15)	Percentage increase in personal use pre- versus postcourse (n = 13)	Percentage increase in professional use pre- versus postcourse (n = 10)
Biofeedback	6.7	69.2*	6.7
Imagery	26.7	44.6*	16.7
Meditation	73.3	13.3	6.7
Exercise	60.0	6.7	23.3
Psychosocial group support	33.3	2.0	−11.7

*P <  .05.

**Figure 1.**
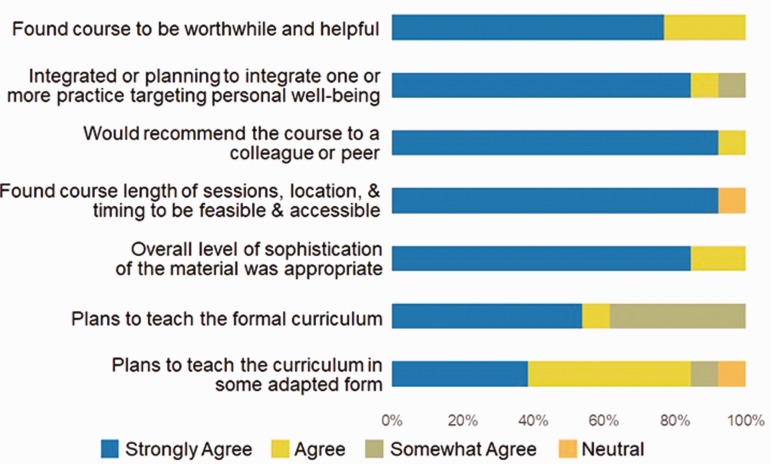
Postcourse Evaluation.

**Figure 2.**
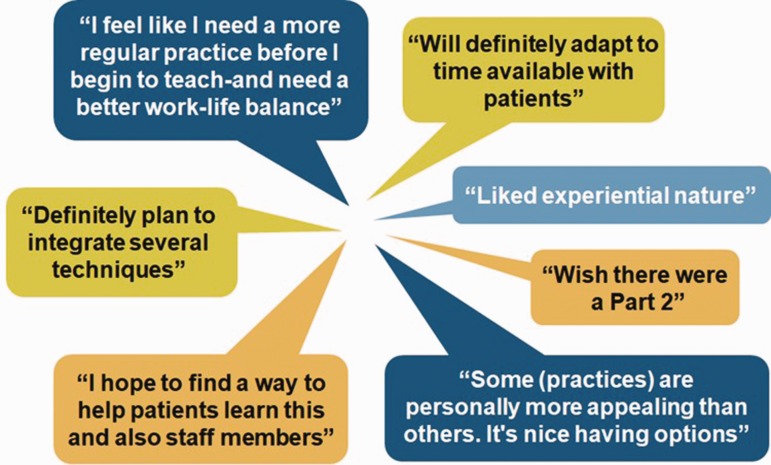
Postcourse Comments.

Postcurricular written evaluations, which included both short answer and multiple-choice responses, showed high levels of satisfaction among participants. There was 100% agreement among participants that they found the course to be worthwhile, that they would recommend the course to a colleague or peer, and that they found the course to be feasible and acceptable. There were no low levels of satisfaction reported (see Figure 1). Short answer responses were positive as reflected in Figure 2. In considering the feasibility of this format, it is notable that 13 out of 15 participants completed the 8-week course.

## Discussion

Initial results from this small cohort indicate that the MORE course has the potential to decrease stress and increase mindfulness among health-care professionals. This is consistent with published literature regarding the positive effects of mind–body medicine modalities for health-care professionals.^15–17^ The feasibility and aggregate efficacy of this curriculum, which is an abbreviated version of the mind–body medicine course designed at the Center for Mind-Body Medicine, would benefit from larger studies using paired pre- and postmeasures.

Given the time constraints on health-care professionals and the need for effective interventions that prevent burnout, improve health, and promote wellness, the preliminary results from this cohort are promising. The increased use of mind–body medicine skills in clinical practice also offers the potential for better outcomes, improved patient safety, and higher patient satisfaction.^7^

The course was well received by the first 2 cohorts of participants. Results support the effectiveness of teaching mind–body medicine skills in this format to health-care professionals. At the end of the course, participants reported increased mindfulness, decreased stress, and increased use of mind–body medicine skills, both personally and professionally. The response to the course was overwhelmingly positive, with most respondents planning to incorporate these skills into their professional lives. These finding support adapting mind–body medicine skills training to the health-care setting.

There are limitations to the extent to which conclusions can be drawn from this small sample of participants.   Results would also be more robust if pre- and postsurveys could be analyzed by individual rather than in aggregate. Participants were not randomized, and they self-selected to attend the course. Instructors shared teaching materials but did not rely upon a set manual. Additional cohorts and the inclusion of longitudinal data would help to define the long-term effects of this intervention. 
